# Multiple trauma including pelvic fracture with multiple arterial embolization: an autopsy case report

**DOI:** 10.1186/s12959-020-00217-y

**Published:** 2020-03-02

**Authors:** Takahito Miyake, Hideshi Okada, Norihide Kanda, Fuminori Yamaji, Haruka Okamoto, Hiroaki Ushikoshi, Kei Noguchi, Hiroyuki Tomita, Shozo Yoshida, Shinji Ogura

**Affiliations:** 1 Advanced Critical Care Center, Gifu University Hospital, 1-1 Yanagido, Gifu, 501-1194 Japan; 2 Department of Tumor Pathology, Gifu University School of Medicine, 1-1 Yanagido, Gifu, 501-1194 Japan

**Keywords:** Pelvic fracture, Arterial embolization, Coagulopathy, Fibrinolysis

## Abstract

**Background:**

Pelvic fracture with high energy trauma has a high mortality rate, especially in men. In addition, severe multiple trauma, major hemorrhage, and administration of red blood cells predict mortality in elderly patients with pelvic fracture. We herein report a rare case in which multiple arterial embolization occurred after pelvic fracture.

**Case presentation:**

An 83-year-old male cyclist was transported to our hospital after being struck by a car. On arrival, he was diagnosed with multiple trauma, including rib fractures with hemothorax, lumbar fractures of the transverse process, and injuries in the right acetabulum, left adrenal gland, and liver. He underwent massive transfusion and transcatheter arterial embolization due to extravasation from the right superior gluteal artery and left adrenal gland. On the second day, owing to right lower leg ischemia, serum creatinine kinase and myoglobin levels were markedly elevated from the reference value; hence, a right above-knee amputation was performed 12 h after the accident. However, both protein levels remained high after amputation, resulting in acute renal injury, which was treated via hemodiafiltration on hospital day 3. In addition, sustained low efficiency hemodialysis and plasma exchange were performed on hospital day 4. Despite these treatments, the patient’s hemodynamics did not improve, and he died on hospital day 8. The autopsy revealed necropsy of the iliopsoas muscles and the digestive tract.

**Conclusions:**

The causes of the patient’s death were considered to be persistent rhabdomyolysis and severe hypotension due to iliopsoas necrosis and peritonitis due to digestive tract necrosis. Multiple arterial embolization caused by consumption coagulopathy associated with multiple trauma may account for severe outcomes in this case.

## Background

Pelvic fracture with high energy trauma has a high mortality rate, especially in men [1]. It can lead to severe multiple trauma or major hemorrhage and may require administration of red blood cells [1, 2]. Its complications, especially in elderly patients, include diabetes, cardiovascular disease, and chronic kidney disease. Chronic diseases sometimes influence the treatment outcomes of patients with pelvic fracture.

We report a case in which multiple arterial embolization occurred after pelvic fracture.

## Case presentation

An 83-year-old male cyclist was transported to our hospital 3 h after being struck by a car. He had a medical history of acute coronary syndrome, for which he had received aspirin. His vital signs were as follows: respiratory rate, 22 breaths per minute; oxygen saturation on room air, 96%; systolic blood pressure, 125/67 mmHg; pulse rate, 88 beats per minute; and body temperature, 36.1 °C. On arrival, the patient was diagnosed with multiple trauma including multiple rib fractures with hemothorax, lumbar fractures of the transverse process, and injuries to the right acetabulum, left adrenal gland, left ureter, and liver. His injury severity score was 22. We also found extravasation of the right superior gluteal artery and left adrenal gland. He was treated with a transfusion of red blood cells, plasma, and platelets, and the ratio was 12:26:30. He was administered 1 g tranexamic acid when he presented in this very acute phase. He also required intubation and transcatheter arterial embolization. There was no evidence of stray embolic materials in the external iliac arteries.

Laboratory test findings (normal ranges in parentheses) on hospital day 1 were as follows: white blood cells, 22.08 × 10^3^/μL (3.3–8.6 × 10^3^/μL); serum hemoglobin, 11.7 mg/dL (13.7–16.8 mg/dL); platelets, 136 × 10^3^/μL (158–348 × 10^3^/μL); serum creatinine kinase, 554 U/L (59–248 U/L); partial thromboplastin time, 37 s (25–38 s); international normalized ratio, 1.31; prothrombin time, 15.8 s (9.8–12.1 s); serum fibrinogen, 107 mg/dL (200–400 mg/dL); serum D-dimer, 155.7 μg/mL (< 1.0 μg/mL); and serum fibrinogen degradation products (FDPs), 305.5 μg/mL (< 5.0 μg/mL). Arterial blood gas analysis revealed mixed acidosis with an elevated serum lactate level (FiO_2_, 0.50; pH, 7.186; pCO_2_, 49.4 Torr; pO_2_ 89.1 Torr; HCO_3_, 18 mmol/L; base excess − 9.9; and lactate, 44 mg/dL).

**Fig. 1:**
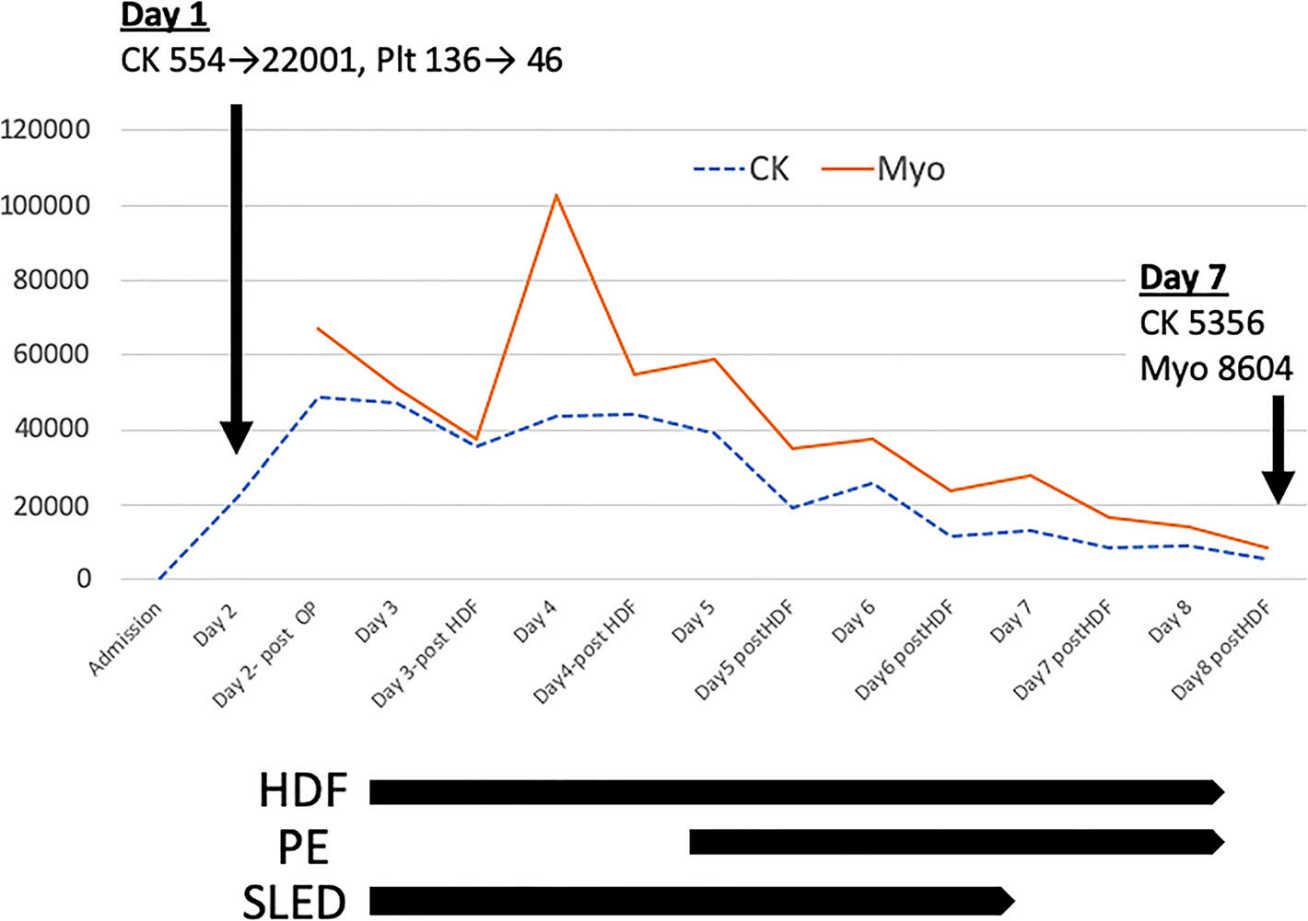
Time course of treatment. CK: creatinine kinase, Plt: platelets, Myo, myoglobin, OP: operation, HDF: hemodiafiltration, PE: plasma exchange, SLED: sustained low efficiency dialysis

On hospital day 2, his serum creatine kinase was markedly elevated to 22,001 U/L. At this point, his right lower leg showed signs of ischemia including lack of a pulse in the right dorsalis pedis artery and cold leg. These findings suggested arterial embolization in the right thigh, and 12 h after the injury, a right above-knee amputation was performed. However, the serum creatine kinase, 44,229 U/L and myoglobin levels, 67,000 ng/mL (< 110 ng/mL), were markedly high, even after the amputation, resulting in acute renal injury, which was treated via hemodiafiltration on hospital day 3. In addition, sustained low efficiency hemodialysis and plasma exchange were performed on hospital day 4. The patient’s creatine kinase and myoglobin levels are plotted against his time course of treatment in Fig. 1. The changes in hemostatic markers are shown in Table 1. Despite these treatments, the patient’s hemodynamic status, which included high catecholamine levels, did not improve, and he died on hospital day 8.

**Table 1:** The Changes of Hemostatic Markers

		Day1	Day2	Day 3	Day 4	Day 5
Platelet	X10^3^/μL	136	46	50	65	74
PT time	seconds	37	34.7	32	32.2	68.6
PT-INR		1.31	1.12	1	1.06	1.07
Fibrinogen	mg/dL	107	199	351	553	692
FDP	μg/mL	305.5	116.9	70.6	26.5	17.7
D-dimer	μg/mL	155.7	51.3	28.7	10.2	5.7

PT: Prothrombin, PT-INR: Prothrombin-International normalized ratio, FDP: Fibrin degradation product

**Fig. 2:**
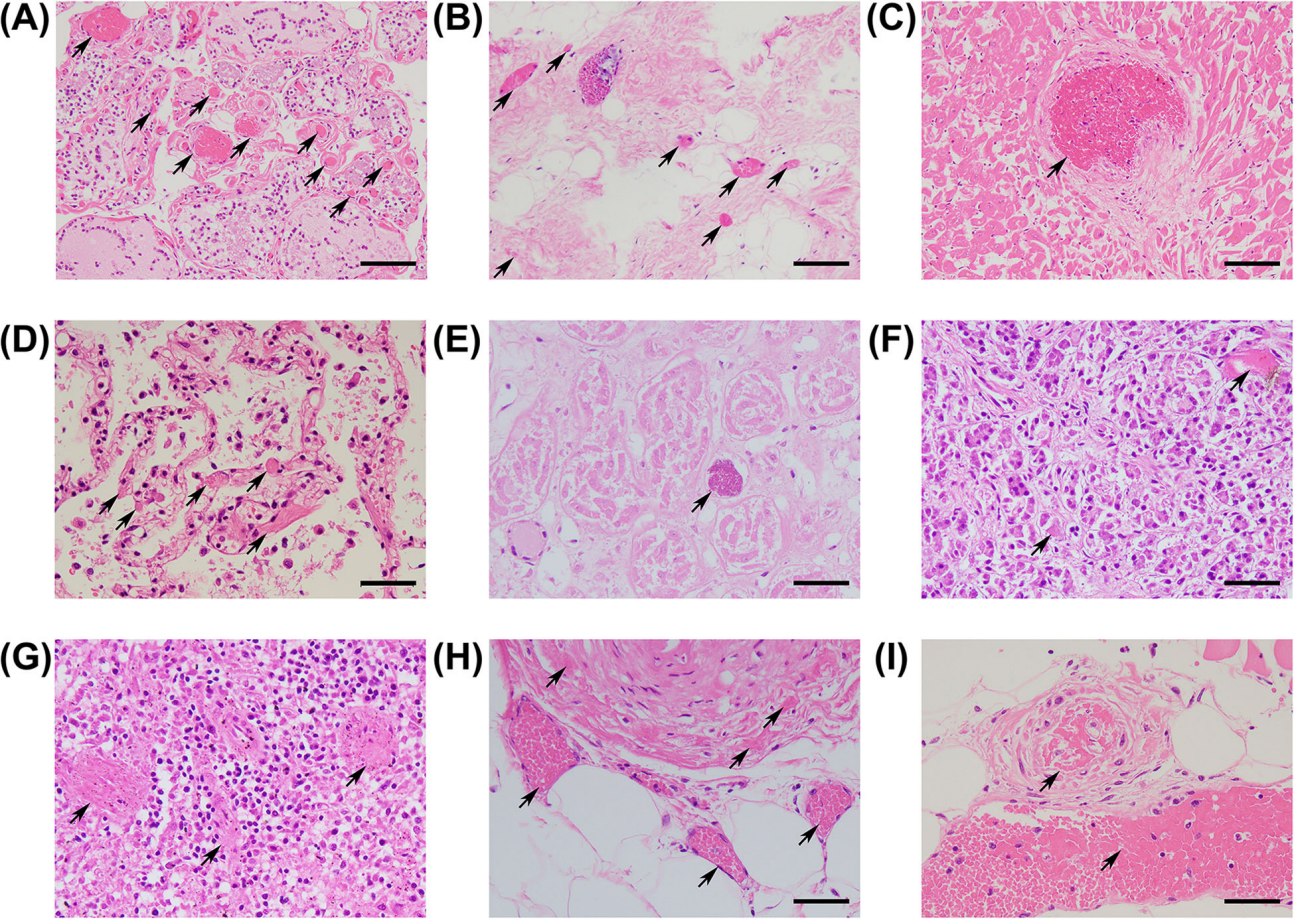
Multiple organ embolization in autopsy specimen. **a** Thyroid gland, (**b**) Left ventricle with atrophic change, (**c**) Left ventricle with non-atrophic change, (**d**) Lung, (**e**) Kidney, (**f**) Pancreas, (**g**) Spleen, (**h**) Transversus Colon, (**i**) Iliopsoas muscle. Arrows indicate thrombus becoming fibrinized. Bars: 50 μm

We performed an autopsy 50 h after his death. A general postmortem examination revealed pulmonary effusion and alveolar hemorrhage. Liver congestion and fatty liver were observed, as were tubular necrosis and nephrosclerosis in the kidney. There were also signs of arteriosclerosis in the aorta, common iliac arteries, and coronary artery. Necrosis was more severe in the left iliopsoas muscle than in the right. Although there were no signs of initial gastrointestinal tract injuries, irreversible ischemic colitis from the descending colon to the rectum was revealed. In addition, thrombus becoming fibrinized was detected in multiple organs in the autopsy specimen. (Fig. 2). This may explain the peritonitis and sepsis, which resulted in continued shock and ultimately death.

## Discussion and conclusions

**Fig. 3:**
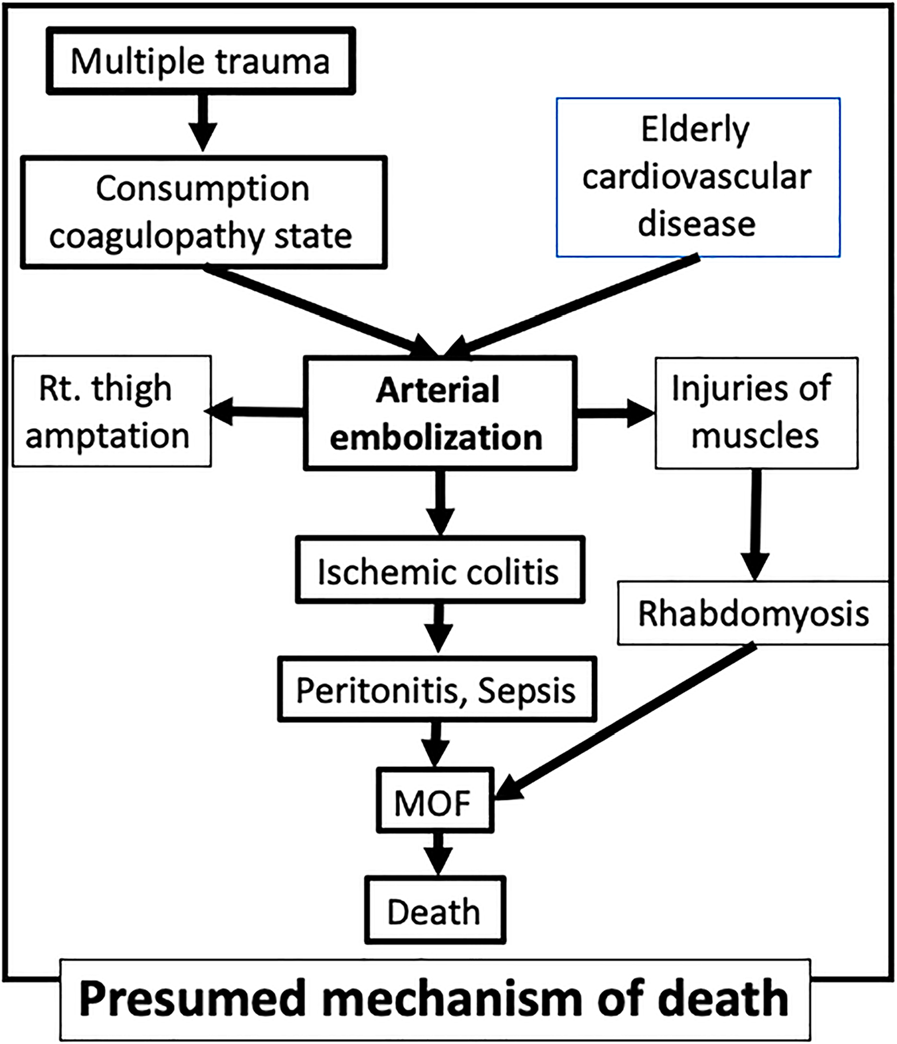
Presumed mechanism of death. Rt: right, MOF: multiple organ failure

The causes of the patient’s death were considered to be persistent rhabdomyolysis due to iliopsoas necrosis and severe hypotension due to digestive tract necrosis and consequent septic peritonitis. Multiple arterial embolization caused by the consumption coagulopathy arising from multiple trauma may be responsible for the acute embolization in multiple organs (Fig. 3). This was a very rare case of pelvic fracture with multiple organ thrombosis.

Acute traumatic coagulopathy develops in approximately 25% of severely injured individuals. It increases the risk of multisystem organ failure substantially and the risk of death by 4-fold [3]. “Trauma-induced coagulopathy” is the generally accepted term for coagulopathy in patients with massive hemorrhage and multiple trauma. It is an endogenous hypocoagulable state unrelated to iatrogenic events. Several distinct but highly integrated mechanisms have been implicated in trauma-induced coagulopathy; these include those resulting in protein C activation, platelet deficit and dysfunction, endothelial involvement, and microparticle formation. The protein C pathway is considered to be a key mediator of traumatic-induced coagulopathy owing to its downstream effects (e.g., thrombin diversion, deactivation of coagulation factors, and de-repression of fibrinolysis) [4].

The most common coagulation disorder after injury is hypercoagulability. Hypofibrinolysis or fibrinolysis shutdown is an integral component of postinjury hypercoagulopathy [3] and is an independent predictor of adverse outcomes after injury, including death [4]. In a previous report, persistent fibrinolysis shutdown independently predicted mortality with an odds ratio of 8.48 (*p* = 0.022) [3]. In reports in which fibrinolysis phenotypes were stratified via thromboelastography (TEG), fibrinolysis shutdown, physiologic fibrinolysis, and hyperfibrinolysis were defined as ≤0.8%, 0.8–3, and > 3% of clot lysis at 30 min after maximum clot strength, respectively [5, 6]. Recent prospective cohort data suggest that severely injured patients more often present with fibrinolysis shutdown than with hyperfibrinolysis or physiologic fibrinolysis [4].

Although we did not perform TEG in this case, we presume that fibrinolysis shutdown was the key mechanism underlying the multiple embolisms in our case. Initial D-dimer and FDP levels were significantly higher than normal, which suggests that the hypercoagulopathy in the very acute phase.

It is very rare that multiple arterial embolization occurs after multiple trauma, while acute traumatic coagulopathy is often caused by severe trauma. This might explain why severely injured patients could die from hypercoagulation during the acute phase of trauma and may account for the paucity of pelvic fractures associated with multiple arterial emboli in the literature.

In this case, the aspirin the patient had received for acute coronary syndrome may have affected the hypercoagulation status, allowing him to survive for several days after his injury. Several studies have investigated the effect of antiplatelet therapy in critically ill patients [7–11]. In the meta-analysis by Du et al., aspirin administration correlated with low hospital mortality rates in critically ill patients [7]. Several observational studies have shown that antiplatelet drugs reduce the levels of biomarkers such as C-reactive protein, soluble CD62P, CD54, and pro-inflammatory cytokines [8, 9]. Other studies, however, found that aspirin did not lower mortality [10] or improve acute lung injuries [11]. Although the role of aspirin in patients in intensive care units is still controversial, aspirin may have some benefits in acute critical situations [7–11].

In addition, this patient’s serum lactate was classified as mixed acidosis. It was thought that the mechanism of this acidosis was mainly from a diabetic origin such as microangiopathy.

In conclusion, severe inflammation caused by severe trauma might cause multiple emboli by inducing hypercoagulation and can sometimes leads to death. Our study highlights the confirmation of hypercoagulation in autopsy. In such situations, anticoagulation therapy may prolong survival.

## Data Availability

The datasets obtained and analyzed in the current study are available from the corresponding author on reasonable request.

## Abbreviations

FDP: fibrinogen degradation products
TEG: thromboelastography
